# Absolute configuration of methyl isoeichlerialactone

**DOI:** 10.1107/S1600536810018295

**Published:** 2010-06-09

**Authors:** Hoong-Kun Fun, Nantiya Joycharat, Supayang Piyawan Voravuthikunchai, Suchada Chantrapromma

**Affiliations:** a X-ray Crystallography Unit, School of Physics, Universiti Sains Malaysia, 11800 USM, Penang, Malaysia; b Natural Products Research Center, Faculty of Science, Prince of Songkla University, Hat-Yai, Songkhla 90112, Thailand; c Crystal Materials Research Unit, Department of Chemistry, Faculty of Science, Prince of Songkla University, Hat-Yai, Songkhla 90112, Thailand

## Abstract

The title compound, C_28_H_44_O_4_·0.56H_2_O, is a co-crystal of methyl isoeichlerialactone monohydrate as the major component and methyl isoeichlerialactone as the minor component in a 0.55778 (3):0.44222 (3) ratio. The conformations of both components are identical except for that of the –COOCH_3_ group of the methyl propanoate side chain on the cyclo­hexane ring which is positionally disordered over two orientations. The mol­ecule of methyl isoeichlerialactone has three fused rings and all rings are *trans*-fused. The two cyclo­hexane rings are in standard chair conformations and the cyclo­pentane ring adopts an envelope conformation. In the crystal, weak C—H⋯O inter­actions link methyl isoeichlerialactone mol­ecules into screw chains along [010]. The crystal structure is further stabilized by O—H⋯O hydrogen bonds and weak C—H⋯O inter­actions.

## Related literature

For details of ring conformations, see: Cremer & Pople (1975 ▶). For bond-length data, see: Allen *et al.* (1987 ▶). For previous studies on 3,4-secodammarane triterpenes in *Aglaia* see: Pointinger *et al.* (2008 ▶); Seger *et al.* (2008 ▶); Joycharat *et al.* (2010 ▶). For related structures, see: Fun *et al.* (2010 ▶); Joycharat *et al.* (2010 ▶). For the stability of the temperature controller used in the data collection, see Cosier & Glazer (1986 ▶).
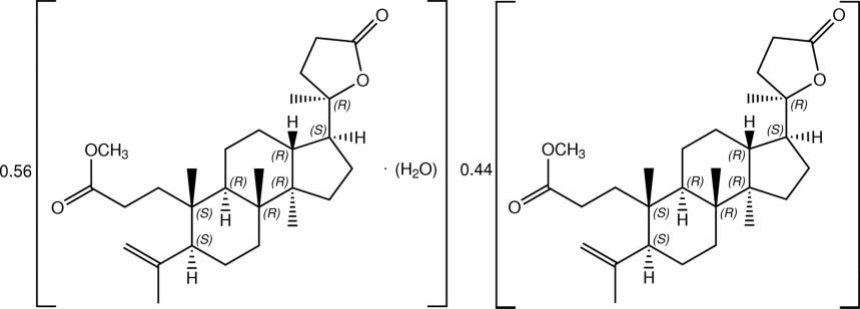


## Experimental

### 

#### Crystal data

C_28_H_44_O_4_·0.56H_2_O
*M* *_r_* = 454.68
Orthorhombic, 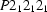

*a* = 7.2246 (2) Å
*b* = 13.3872 (4) Å
*c* = 26.1898 (8) Å
*V* = 2533.00 (13) Å^3^
*Z* = 4
Cu *K*α radiation
μ = 0.62 mm^−1^
*T* = 100 K
0.34 × 0.23 × 0.05 mm

#### Data collection

Bruker APEXII DUO CCD area-detector diffractometer
Absorption correction: multi-scan (*SADABS*; Bruker, 2009 ▶) *T*_min_ = 0.818, *T*_max_ = 0.969
52930 measured reflections
3968 independent reflections
3522 reflections with *I* > 2σ(*I*)
*R*_int_ = 0.044

#### Refinement

*R*[*F* ^2^ > 2σ(*F* ^2^)] = 0.036
*wR*(*F* ^2^) = 0.099
*S* = 1.05
3968 reflections
327 parameters
H-atom parameters constrained
Δρ_max_ = 0.18 e Å^−3^
Δρ_min_ = −0.19 e Å^−3^
Absolute structure: Flack (1983 ▶), 1634 Friedel pairs
Flack parameter: 0.0 (2)

### 

Data collection: *APEX2* (Bruker, 2009 ▶); cell refinement: *SAINT* (Bruker, 2009 ▶); data reduction: *SAINT*; program(s) used to solve structure: *SHELXTL* (Sheldrick, 2008 ▶); program(s) used to refine structure: *SHELXTL*; molecular graphics: *SHELXTL*; software used to prepare material for publication: *SHELXTL* and *PLATON* (Spek, 2009 ▶).

## Supplementary Material

Crystal structure: contains datablocks global, I. DOI: 10.1107/S1600536810018295/sj5002sup1.cif

Structure factors: contains datablocks I. DOI: 10.1107/S1600536810018295/sj5002Isup2.hkl

Additional supplementary materials:  crystallographic information; 3D view; checkCIF report

## Figures and Tables

**Table 1:** Hydrogen-bond geometry (Å, °)

*D*—H⋯*A*	*D*—H	H⋯*A*	*D*⋯*A*	*D*—H⋯*A*
O1*W*—H1*W*1⋯O4^i^	1.06	1.94	2.912 (4)	151
C2—H2*A*⋯O4^ii^	0.97	2.45	3.305 (3)	146
C12—H12*B*⋯O3	0.97	2.58	3.154 (2)	118

Symmetry codes: (i) 
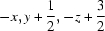
; (ii) 
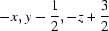
.

**Fig. 1.**
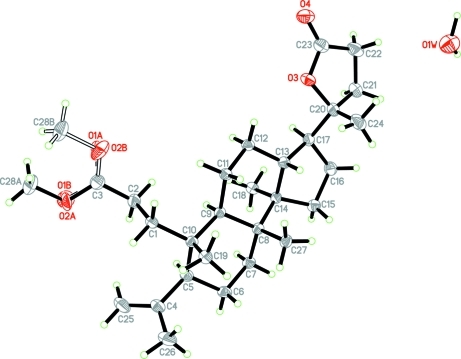
The molecular structure of the title compound, with 40% probability displacement ellipsoids and the atom-numbering scheme. Open bonds show the minor component.

**Fig. 2.**
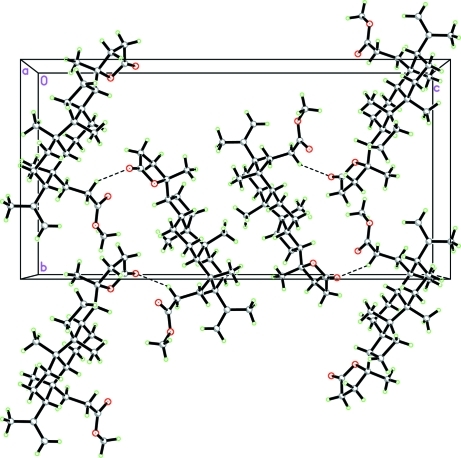
The crystal packing of the major component of the title compound viewed along the a axis, showing screw chains along the [010] direction. Weak C—H···O interactions are shown as dashed lines. Water molecules and atoms of the minor disorder component were omitted for clarity.

## supplementary crystallographic information

### Comment

The accumulation of two different stereochemical types of 3,4-secodammarane triterpenes characterised by linking the tetrahydrofuran ring of the side chain to the cyclopentane ring of the sterane skeleton either towards 20*S* or 20*R* configuration in *Aglaia* species has recently been described (Pointinger *et al.*, 2008; Seger *et al.*, 2008). Recently, we have confirmed an absolute configuration of the antiphytopathogenic fungal agent, isoeichlerialactone, isolated from the ethanolic seed extract of *Aglaia forbesii* King, family Meliaceae collected in Thailand (Fun *et al.*, 2010; Joycharat *et al*., 2010). Moreover, from the seed extract of *Aglaia forbesii*, the title compound, the corresponding ester of isoeichlerialactone, was isolated as a minor component (Joycharat *et al.*,  2010). Herein we reported the absolute configuration of the title seco-dammarane triterpenoid namely methyl isoeichlerialactone [systematic name: methyl 3-((3*S*,3a*R*,5a*R*,6*S*,7*S*,9a*R*, 9b*R*)-6,9a,9b-trimethyl-3-((*R*)-2-methyl-5-oxotetrahydrofuran-2-yl)- 7-(prop-1-en-2-yl)dodecahydro-1*H*-cyclopenta[*a*]naphthalen-6-yl) propanoate], (I). Its absolute configuration was determined by making use of the anomalous scattering of Cu Kα*X*-radiation and the Flack parameter is 0.0 (2).

The asymmetric unit of the title compound (Fig. 1) consists of methyl isoeichlerialactone monohydrate as the major component and methyl isoeichlerialactone as the minor component. The refined site-occupancy ratio of the major and minor components is 0.55778 (3)/0.44222 (3). The conformations and absolute configuration of both components are identical except for that of the COOCH_3_ group of the methyl propanoate side chain (C1–C3/O1–O2/C28) on the cyclohexane ring is positionally disordered over two positions [*A* and *B*] with the occupancy ratio given above (Fig. 1). The molecule of methyl isoeichlerialactone, has three fused rings and all rings are  *trans*-fused. The two cyclohexane rings are in standard chair conformations. The cyclopentane (C13–C17) adopts an envelope conformation with the puckered C14 atom having the maximum deviation of 0.259 (2) Å, Q = 0.420 (2) Å and θ = 202.9 (3)° whereas the furan ring (C20–C23/O3) is twisted with the twisted C20 and C21 atoms having the deviation of -0.144 (2) and 0.162 (3) Å, respectively from the C22/C23/O3 plane with Q = 0.259 (3) Å and θ = 64.2 (5)° (Cremer & Pople, 1975). Atoms C2, C3, C28, O1 and O2 of the methyl propanoate group are lie almost on the same plane with the *r.m.s*. deviation 0.0138 (2) and 0.0296 (2) Å for major and minor component, respectively and the torsion angles C28A–O2A–C3–O1A = -4.2 (16)° whereas C28B–O2B–C3–O1B = -4(3)°. The orientation of this disordered side chain is described by the torsion angles C10–C1–C2–C3 = -175.99 (17)°, C1–C2–C3–O1A = 88.7 (12)° and  C1–C2–C3–O2A = -92.9 (5)°; C1–C2–C3–O1B = -96.4 (6) and C1–C2–C3–O2B = 75.3 (14)°. The bond angles around C4 and C25 atoms are indicative of *sp*^2^ hybridization for these atoms and the bond length of 1.398 (3) Å confirmed the C4 ═C25 bond. The configurations at atoms C5, C8, C9, C10, C13, C14, C17 and C20 are in *S*, *R*, *R*, *S*, *R*, *R*, *S* and *R*, respectively. The bond distances have normal values (Allen *et al.*, 1987) and comparable with the closely related compound (Fun *et al.*, 2010).

The crystal packing of the major component is shown in Fig. 2, with the methyl isoeichlerialactone molecules being linked through weak C—H···O interactions (Table 1) into screw chains along the *b* axis. The packing of the minor component is same as that of the major component. The crystal is stabilized by intermolecular O—H···O hydrogen bonds and weak C—H···O interactions (Table 1).

### Experimental

The seeds of *Aglaia forbesii* (48 g) were air-dried, ground, and exhaustively extracted with EtOH (3 *x* 500 *mL*) at room temperature. The combined extracts were concentrated under reduced pressure to afford a brown extract (5.7 g) which was resuspended in a mixture of MeOH and water and then extracted with n-hexane, CH_2_Cl_2_, and BuOH, successively. The CH_2_Cl_2_ fraction (1.87 g) was applied to column chromatography (CC) over silica gel (Merck, 0.063-0.200 mm) using gradient elution from 0% to 100% acetone in CH_2_Cl_2_, and finally washed down with MeOH. The fraction eluted with 20% acetone in CH_2_Cl_2_ was further subjected to repeated silica gel column chromatography ((i) CC with Hexane/Acetone, 100:0 to 0:100 and (ii) CC with CH_2_Cl_2_/EtOAc, 98:2, v/v) to afford the title compound (3 mg). Colorless plate-shaped single crystals of the title compound suitable for *X*-ray structure determination were recrystallized from EtOH after several days. ^1^H NMR and ^13^ C NMR spectral data (Joycharat *et al.*, 2010) were consistent with the *X*-ray structure.

### Refinement

All H atoms were placed in calculated positions with d(C—H) = 0.98 Å for CH; 0.97 Å for CH_2_ and 0.96 Å for CH_3_ atoms. The *U*_iso_ values were constrained to be 1.5*U*_eq_ of the carrier atom for methyl H atoms and 1.2*U*_eq_ for the remaining H atoms. A rotating group model was used for the methyl groups. The highest residual electron density peak is located at 0.99 Å from H25B and the deepest hole is located at 0.21 Å from C28B. 1634 Friedel pairs were used to determine the absolute configuration.

### Crystal data

**Table:**

C_28_H_44_O_4_·0.56H_2_O	*F*(000) = 998.1
*M**_r_* = 454.68	*D*_x_ = 1.192 Mg m^−^^3^
Orthorhombic, *P*2_1_2_1_2_1_	Cu *K*α radiation, λ = 1.54178 Å
Hall symbol: P 2ac 2ab	Cell parameters from 3968 reflections
*a* = 7.2246 (2) Å	θ = 3.4–63.0°
*b* = 13.3872 (4) Å	µ = 0.62 mm^−^^1^
*c* = 26.1898 (8) Å	*T* = 100 K
*V* = 2533.00 (13) Å^3^	Plate, colorless
*Z* = 4	0.34 × 0.23 × 0.05 mm

### Data collection

**Table:**

Bruker APEXII DUO CCD area-detector diffractometer	3968 independent reflections
Radiation source: sealed tube	3522 reflections with *I* > 2σ(*I*)
graphite	*R*_int_ = 0.044
φ and ω scans	θ_max_ = 63.0°, θ_min_ = 3.4°
Absorption correction: multi-scan (*SADABS*; Bruker, 2009)	*h* = −8→7
*T*_min_ = 0.818, *T*_max_ = 0.969	*k* = −15→15
52930 measured reflections	*l* = −29→30

### Refinement

**Table:**

Refinement on *F*^2^	Secondary atom site location: difference Fourier map
Least-squares matrix: full	Hydrogen site location: inferred from neighbouring sites
*R*[*F*^2^ > 2σ(*F*^2^)] = 0.036	H-atom parameters constrained
*wR*(*F*^2^) = 0.099	*w* = 1/[σ^2^(*F*_o_^2^) + (0.0559*P*)^2^ + 0.5383*P*] where *P* = (*F*_o_^2^ + 2*F*_c_^2^)/3
*S* = 1.05	(Δ/σ)_max_ = 0.001
3968 reflections	Δρ_max_ = 0.18 e Å^−^^3^
327 parameters	Δρ_min_ = −0.19 e Å^−^^3^
0 restraints	Absolute structure: Flack (1983), 1634 Friedel pairs
Primary atom site location: structure-invariant direct methods	Flack parameter: 0.0 (2)

### Special details

**Table:**

Experimental. The crystal was placed in the cold stream of an Oxford Cryosystems Cobra open-flow nitrogen cryostat (Cosier & Glazer, 1986) operating at 100.0 (1) K.
Geometry. All esds (except the esd in the dihedral angle between two l.s. planes) are estimated using the full covariance matrix. The cell esds are taken into account individually in the estimation of esds in distances, angles and torsion angles; correlations between esds in cell parameters are only used when they are defined by crystal symmetry. An approximate (isotropic) treatment of cell esds is used for estimating esds involving l.s. planes.
Refinement. Refinement of F^2^ against ALL reflections. The weighted R-factor wR and goodness of fit S are based on F^2^, conventional R-factors R are based on F, with F set to zero for negative F^2^. The threshold expression of F^2^ > 2sigma(F^2^) is used only for calculating R-factors(gt) etc. and is not relevant to the choice of reflections for refinement. R-factors based on F^2^ are statistically about twice as large as those based on F, and R- factors based on ALL data will be even larger.

### Fractional atomic coordinates and isotropic or equivalent isotropic displacement parameters (Å^2^)

**Table:**

	*x*	*y*	*z*	*U*_iso_*/*U*_eq_	Occ. (<1)
O1A	−0.273 (2)	−0.1142 (10)	0.8174 (8)	0.044 (3)	0.558 (3)
O2A	−0.1434 (13)	−0.2556 (6)	0.8441 (4)	0.050 (4)	0.558 (3)
O1B	−0.1464 (14)	−0.2552 (8)	0.8408 (5)	0.044 (4)	0.442 (3)
O2B	−0.286 (3)	−0.1069 (15)	0.8266 (10)	0.051 (5)	0.442 (3)
O3	0.0321 (2)	0.44352 (10)	0.80263 (5)	0.0326 (4)	
O4	−0.1588 (2)	0.51204 (12)	0.74616 (6)	0.0430 (4)	
C1	−0.0153 (3)	−0.07230 (15)	0.91294 (7)	0.0241 (4)	
H1B	−0.0341	−0.1316	0.9336	0.029*	
H1A	−0.1305	−0.0351	0.9133	0.029*	
C2	0.0210 (3)	−0.10586 (17)	0.85799 (7)	0.0318 (5)	
H2A	0.0452	−0.0482	0.8366	0.038*	
H2B	0.1287	−0.1491	0.8569	0.038*	
C3	−0.1439 (3)	−0.16044 (19)	0.83871 (8)	0.0349 (5)	
C4	0.2820 (3)	−0.16680 (16)	0.97910 (8)	0.0302 (5)	
C5	0.3125 (3)	−0.07180 (15)	0.94891 (8)	0.0259 (5)	
H5A	0.3543	−0.0933	0.9151	0.031*	
C6	0.4708 (3)	−0.00839 (15)	0.97075 (8)	0.0289 (5)	
H6A	0.4339	0.0184	1.0036	0.035*	
H6B	0.5786	−0.0504	0.9760	0.035*	
C7	0.5214 (3)	0.07702 (15)	0.93534 (7)	0.0263 (5)	
H7A	0.5692	0.0495	0.9037	0.032*	
H7B	0.6196	0.1158	0.9510	0.032*	
C8	0.3586 (3)	0.14722 (15)	0.92282 (7)	0.0221 (4)	
C9	0.1912 (3)	0.08197 (14)	0.90423 (7)	0.0205 (4)	
H9A	0.2350	0.0505	0.8727	0.025*	
C10	0.1343 (3)	−0.00757 (15)	0.93938 (7)	0.0224 (4)	
C11	0.0263 (3)	0.14707 (15)	0.88736 (7)	0.0247 (5)	
H11A	−0.0693	0.1041	0.8734	0.030*	
H11B	−0.0248	0.1803	0.9171	0.030*	
C12	0.0774 (3)	0.22616 (15)	0.84740 (7)	0.0259 (5)	
H12A	0.1084	0.1938	0.8154	0.031*	
H12B	−0.0276	0.2698	0.8415	0.031*	
C13	0.2412 (3)	0.28711 (15)	0.86592 (7)	0.0239 (5)	
H13A	0.2034	0.3184	0.8981	0.029*	
C14	0.4102 (3)	0.22061 (15)	0.87820 (7)	0.0224 (5)	
C15	0.5590 (3)	0.30013 (15)	0.88955 (8)	0.0289 (5)	
H15A	0.6820	0.2731	0.8840	0.035*	
H15B	0.5498	0.3230	0.9246	0.035*	
C16	0.5195 (3)	0.38659 (16)	0.85200 (8)	0.0337 (5)	
H16A	0.6069	0.3853	0.8239	0.040*	
H16B	0.5299	0.4505	0.8693	0.040*	
C17	0.3182 (3)	0.37058 (15)	0.83198 (7)	0.0267 (5)	
H17A	0.3272	0.3441	0.7972	0.032*	
C18	0.4771 (3)	0.16521 (15)	0.82951 (7)	0.0270 (5)	
H18A	0.5017	0.2129	0.8030	0.041*	
H18B	0.3827	0.1197	0.8183	0.041*	
H18C	0.5881	0.1287	0.8371	0.041*	
C19	0.0426 (3)	0.02362 (15)	0.99005 (7)	0.0256 (5)	
H19A	−0.0180	−0.0331	1.0050	0.038*	
H19B	−0.0468	0.0752	0.9836	0.038*	
H19C	0.1354	0.0482	1.0131	0.038*	
C20	0.2067 (3)	0.46699 (16)	0.82936 (8)	0.0317 (5)	
C21	0.2973 (4)	0.54557 (17)	0.79445 (9)	0.0412 (6)	
H21A	0.3644	0.5949	0.8143	0.049*	
H21B	0.3819	0.5144	0.7705	0.049*	
C22	0.1347 (4)	0.59283 (19)	0.76687 (9)	0.0447 (6)	
H22A	0.0959	0.6538	0.7838	0.054*	
H22B	0.1663	0.6081	0.7317	0.054*	
C23	−0.0139 (3)	0.51499 (17)	0.76943 (7)	0.0346 (5)	
C24	0.1526 (4)	0.51003 (18)	0.88086 (8)	0.0483 (7)	
H24A	0.0661	0.4661	0.8973	0.073*	
H24B	0.0965	0.5744	0.8761	0.073*	
H24C	0.2610	0.5169	0.9018	0.073*	
C25	0.2746 (3)	−0.25788 (19)	0.95303 (10)	0.0453 (6)	
H25A	0.2633	−0.3173	0.9712	0.054*	
H25B	0.2809	−0.2590	0.9176	0.054*	
C26	0.2721 (3)	−0.16537 (19)	1.03380 (9)	0.0429 (6)	
H26A	0.2412	−0.2308	1.0461	0.064*	
H26B	0.1789	−0.1187	1.0444	0.064*	
H26C	0.3898	−0.1456	1.0475	0.064*	
C27	0.3098 (3)	0.20673 (15)	0.97170 (7)	0.0264 (5)	
H27A	0.1833	0.2283	0.9700	0.040*	
H27B	0.3894	0.2640	0.9743	0.040*	
H27C	0.3266	0.1648	1.0011	0.040*	
C28A	−0.3009 (7)	−0.3076 (4)	0.82808 (17)	0.0485 (9)	0.558 (3)
H28A	−0.2859	−0.3774	0.8353	0.073*	0.558 (3)
H28B	−0.3175	−0.2984	0.7920	0.073*	0.558 (3)
H28C	−0.4074	−0.2826	0.8459	0.073*	0.558 (3)
C28B	−0.4627 (8)	−0.1624 (5)	0.8110 (2)	0.0485 (9)	0.442 (3)
H28D	−0.5465	−0.1166	0.7949	0.073*	0.442 (3)
H28E	−0.5202	−0.1905	0.8407	0.073*	0.442 (3)
H28F	−0.4319	−0.2149	0.7875	0.073*	0.442 (3)
O1W	0.3649 (5)	0.8328 (3)	0.78098 (12)	0.0633 (11)	0.558 (3)
H1W1	0.2851	0.8846	0.7596	0.095*	0.558 (3)
H2W1	0.4764	0.8450	0.7786	0.095*	0.558 (3)

### Atomic displacement parameters (Å^2^)

**Table:**

	*U*^11^	*U*^22^	*U*^33^	*U*^12^	*U*^13^	*U*^23^
O1A	0.063 (6)	0.024 (4)	0.043 (5)	−0.004 (3)	−0.021 (4)	−0.002 (4)
O2A	0.080 (6)	0.034 (6)	0.036 (4)	−0.034 (3)	−0.006 (4)	0.010 (3)
O1B	0.039 (6)	0.035 (8)	0.056 (7)	0.020 (5)	−0.002 (4)	−0.017 (5)
O2B	0.028 (5)	0.069 (8)	0.057 (10)	−0.002 (4)	−0.010 (4)	−0.019 (4)
O3	0.0336 (9)	0.0289 (8)	0.0354 (8)	0.0033 (7)	−0.0003 (7)	0.0093 (7)
O4	0.0472 (11)	0.0473 (10)	0.0344 (8)	0.0120 (8)	−0.0045 (8)	0.0014 (8)
C1	0.0210 (11)	0.0223 (11)	0.0289 (10)	0.0008 (8)	0.0004 (9)	0.0052 (8)
C2	0.0377 (13)	0.0289 (12)	0.0289 (11)	−0.0039 (10)	−0.0012 (10)	−0.0018 (9)
C3	0.0434 (16)	0.0353 (16)	0.0261 (11)	0.0009 (12)	−0.0010 (11)	−0.0051 (12)
C4	0.0179 (11)	0.0294 (13)	0.0434 (12)	0.0039 (9)	−0.0001 (9)	0.0099 (10)
C5	0.0228 (11)	0.0259 (12)	0.0291 (10)	0.0037 (9)	0.0015 (9)	0.0032 (9)
C6	0.0218 (11)	0.0319 (13)	0.0330 (10)	0.0039 (9)	−0.0037 (9)	0.0065 (10)
C7	0.0187 (11)	0.0319 (12)	0.0284 (10)	−0.0018 (9)	−0.0027 (9)	0.0040 (9)
C8	0.0181 (11)	0.0244 (12)	0.0239 (9)	0.0008 (8)	0.0003 (8)	−0.0004 (8)
C9	0.0183 (11)	0.0216 (11)	0.0218 (9)	0.0015 (8)	−0.0003 (8)	−0.0003 (8)
C10	0.0209 (11)	0.0226 (12)	0.0238 (9)	0.0024 (8)	−0.0007 (8)	0.0002 (9)
C11	0.0194 (11)	0.0252 (12)	0.0294 (10)	−0.0009 (8)	−0.0025 (9)	0.0033 (9)
C12	0.0221 (11)	0.0267 (12)	0.0291 (10)	0.0011 (8)	−0.0032 (8)	0.0037 (9)
C13	0.0240 (11)	0.0238 (12)	0.0240 (10)	0.0019 (8)	0.0007 (8)	−0.0011 (9)
C14	0.0189 (11)	0.0226 (11)	0.0257 (10)	−0.0006 (8)	−0.0009 (8)	0.0001 (9)
C15	0.0241 (12)	0.0327 (12)	0.0299 (11)	−0.0021 (9)	−0.0001 (8)	0.0022 (9)
C16	0.0338 (13)	0.0300 (13)	0.0372 (12)	−0.0067 (10)	−0.0007 (10)	0.0047 (10)
C17	0.0293 (12)	0.0235 (11)	0.0274 (10)	−0.0012 (9)	0.0002 (9)	−0.0004 (9)
C18	0.0253 (12)	0.0285 (12)	0.0273 (10)	0.0020 (9)	0.0053 (9)	0.0031 (9)
C19	0.0230 (11)	0.0262 (11)	0.0277 (10)	−0.0001 (9)	0.0027 (9)	0.0011 (9)
C20	0.0390 (13)	0.0245 (12)	0.0316 (11)	−0.0026 (9)	−0.0025 (10)	0.0024 (9)
C21	0.0503 (15)	0.0284 (13)	0.0450 (13)	−0.0039 (11)	−0.0042 (12)	0.0064 (11)
C22	0.0555 (17)	0.0375 (15)	0.0410 (13)	0.0027 (11)	−0.0003 (12)	0.0145 (11)
C23	0.0438 (15)	0.0349 (14)	0.0252 (10)	0.0083 (11)	0.0036 (11)	0.0010 (10)
C24	0.078 (2)	0.0308 (14)	0.0359 (12)	0.0161 (13)	−0.0014 (12)	−0.0034 (11)
C25	0.0457 (15)	0.0310 (14)	0.0593 (15)	0.0032 (11)	−0.0005 (12)	0.0161 (12)
C26	0.0327 (14)	0.0439 (15)	0.0521 (14)	0.0036 (11)	−0.0031 (11)	0.0203 (13)
C27	0.0246 (12)	0.0276 (12)	0.0272 (10)	−0.0050 (9)	−0.0016 (9)	−0.0001 (9)
C28A	0.039 (2)	0.059 (2)	0.047 (2)	−0.0197 (18)	0.0002 (17)	−0.0083 (18)
C28B	0.039 (2)	0.059 (2)	0.047 (2)	−0.0197 (18)	0.0002 (17)	−0.0083 (18)
O1W	0.048 (2)	0.079 (3)	0.063 (2)	0.0069 (18)	0.0034 (18)	−0.0044 (19)

### Geometric parameters (Å, °)

**Table:**

O1A—C3	1.253 (14)	C14—C15	1.542 (3)
O2A—C3	1.282 (9)	C14—C18	1.552 (3)
O2A—C28A	1.398 (11)	C15—C16	1.545 (3)
O1B—C3	1.270 (11)	C15—H15A	0.9700
O2B—C3	1.291 (19)	C15—H15B	0.9700
O2B—C28B	1.533 (19)	C16—C17	1.561 (3)
O3—C23	1.335 (3)	C16—H16A	0.9700
O3—C20	1.477 (3)	C16—H16B	0.9700
O4—C23	1.212 (3)	C17—C20	1.523 (3)
C1—C2	1.530 (3)	C17—H17A	0.9800
C1—C10	1.549 (3)	C18—H18A	0.9600
C1—H1B	0.9700	C18—H18B	0.9600
C1—H1A	0.9700	C18—H18C	0.9600
C2—C3	1.486 (3)	C19—H19A	0.9600
C2—H2A	0.9700	C19—H19B	0.9600
C2—H2B	0.9700	C19—H19C	0.9600
C4—C25	1.398 (3)	C20—C24	1.518 (3)
C4—C26	1.434 (3)	C20—C21	1.540 (3)
C4—C5	1.514 (3)	C21—C22	1.517 (3)
C5—C6	1.535 (3)	C21—H21A	0.9700
C5—C10	1.568 (3)	C21—H21B	0.9700
C5—H5A	0.9800	C22—C23	1.498 (3)
C6—C7	1.517 (3)	C22—H22A	0.9700
C6—H6A	0.9700	C22—H22B	0.9700
C6—H6B	0.9700	C24—H24A	0.9600
C7—C8	1.540 (3)	C24—H24B	0.9600
C7—H7A	0.9700	C24—H24C	0.9600
C7—H7B	0.9700	C25—H25A	0.9300
C8—C27	1.548 (3)	C25—H25B	0.9300
C8—C9	1.570 (3)	C26—H26A	0.9600
C8—C14	1.572 (3)	C26—H26B	0.9600
C9—C11	1.541 (3)	C26—H26C	0.9600
C9—C10	1.566 (3)	C27—H27A	0.9600
C9—H9A	0.9800	C27—H27B	0.9600
C10—C19	1.541 (3)	C27—H27C	0.9600
C11—C12	1.534 (3)	C28A—H28A	0.9600
C11—H11A	0.9700	C28A—H28B	0.9600
C11—H11B	0.9700	C28A—H28C	0.9600
C12—C13	1.517 (3)	C28B—H28D	0.9600
C12—H12A	0.9700	C28B—H28E	0.9600
C12—H12B	0.9700	C28B—H28F	0.9600
C13—C17	1.532 (3)	O1W—H1W1	1.0623
C13—C14	1.545 (3)	O1W—H2W1	0.8240
C13—H13A	0.9800		
			
C3—O2A—C28A	117.3 (7)	C15—C14—C8	116.96 (15)
C3—O2B—C28B	117.3 (14)	C13—C14—C8	109.14 (15)
C23—O3—C20	111.64 (16)	C18—C14—C8	112.70 (16)
C2—C1—C10	117.72 (16)	C14—C15—C16	105.42 (16)
C2—C1—H1B	107.9	C14—C15—H15A	110.7
C10—C1—H1B	107.9	C16—C15—H15A	110.7
C2—C1—H1A	107.9	C14—C15—H15B	110.7
C10—C1—H1A	107.9	C16—C15—H15B	110.7
H1B—C1—H1A	107.2	H15A—C15—H15B	108.8
C3—C2—C1	109.05 (18)	C15—C16—C17	106.42 (17)
C3—C2—H2A	109.9	C15—C16—H16A	110.4
C1—C2—H2A	109.9	C17—C16—H16A	110.4
C3—C2—H2B	109.9	C15—C16—H16B	110.4
C1—C2—H2B	109.9	C17—C16—H16B	110.4
H2A—C2—H2B	108.3	H16A—C16—H16B	108.6
O1A—C3—O1B	120.2 (9)	C20—C17—C13	116.89 (17)
O1A—C3—O2A	122.9 (8)	C20—C17—C16	113.05 (18)
O1B—C3—O2B	123.7 (10)	C13—C17—C16	104.10 (16)
O2A—C3—O2B	125.6 (9)	C20—C17—H17A	107.4
O1A—C3—C2	120.4 (7)	C13—C17—H17A	107.4
O1B—C3—C2	119.2 (6)	C16—C17—H17A	107.4
O2A—C3—C2	116.7 (5)	C14—C18—H18A	109.5
O2B—C3—C2	116.6 (9)	C14—C18—H18B	109.5
C25—C4—C26	119.8 (2)	H18A—C18—H18B	109.5
C25—C4—C5	118.88 (19)	C14—C18—H18C	109.5
C26—C4—C5	121.2 (2)	H18A—C18—H18C	109.5
C4—C5—C6	112.25 (16)	H18B—C18—H18C	109.5
C4—C5—C10	115.08 (16)	C10—C19—H19A	109.5
C6—C5—C10	111.58 (16)	C10—C19—H19B	109.5
C4—C5—H5A	105.7	H19A—C19—H19B	109.5
C6—C5—H5A	105.7	C10—C19—H19C	109.5
C10—C5—H5A	105.7	H19A—C19—H19C	109.5
C7—C6—C5	111.62 (16)	H19B—C19—H19C	109.5
C7—C6—H6A	109.3	O3—C20—C24	106.38 (18)
C5—C6—H6A	109.3	O3—C20—C17	107.03 (16)
C7—C6—H6B	109.3	C24—C20—C17	114.70 (18)
C5—C6—H6B	109.3	O3—C20—C21	103.13 (16)
H6A—C6—H6B	108.0	C24—C20—C21	112.19 (19)
C6—C7—C8	113.96 (16)	C17—C20—C21	112.40 (18)
C6—C7—H7A	108.8	C22—C21—C20	103.79 (19)
C8—C7—H7A	108.8	C22—C21—H21A	111.0
C6—C7—H7B	108.8	C20—C21—H21A	111.0
C8—C7—H7B	108.8	C22—C21—H21B	111.0
H7A—C7—H7B	107.7	C20—C21—H21B	111.0
C7—C8—C27	108.17 (15)	H21A—C21—H21B	109.0
C7—C8—C9	108.34 (15)	C23—C22—C21	104.10 (18)
C27—C8—C9	111.55 (16)	C23—C22—H22A	110.9
C7—C8—C14	111.03 (16)	C21—C22—H22A	110.9
C27—C8—C14	110.31 (16)	C23—C22—H22B	110.9
C9—C8—C14	107.44 (14)	C21—C22—H22B	110.9
C11—C9—C10	113.48 (15)	H22A—C22—H22B	109.0
C11—C9—C8	111.72 (15)	O4—C23—O3	121.3 (2)
C10—C9—C8	116.48 (15)	O4—C23—C22	128.3 (2)
C11—C9—H9A	104.6	O3—C23—C22	110.47 (19)
C10—C9—H9A	104.6	C20—C24—H24A	109.5
C8—C9—H9A	104.6	C20—C24—H24B	109.5
C19—C10—C1	103.69 (15)	H24A—C24—H24B	109.5
C19—C10—C9	114.31 (16)	C20—C24—H24C	109.5
C1—C10—C9	110.40 (15)	H24A—C24—H24C	109.5
C19—C10—C5	111.38 (15)	H24B—C24—H24C	109.5
C1—C10—C5	109.72 (15)	C4—C25—H25A	120.0
C9—C10—C5	107.31 (15)	C4—C25—H25B	120.0
C12—C11—C9	113.57 (16)	H25A—C25—H25B	120.0
C12—C11—H11A	108.9	C4—C26—H26A	109.5
C9—C11—H11A	108.9	C4—C26—H26B	109.5
C12—C11—H11B	108.9	H26A—C26—H26B	109.5
C9—C11—H11B	108.9	C4—C26—H26C	109.5
H11A—C11—H11B	107.7	H26A—C26—H26C	109.5
C13—C12—C11	109.93 (16)	H26B—C26—H26C	109.5
C13—C12—H12A	109.7	C8—C27—H27A	109.5
C11—C12—H12A	109.7	C8—C27—H27B	109.5
C13—C12—H12B	109.7	H27A—C27—H27B	109.5
C11—C12—H12B	109.7	C8—C27—H27C	109.5
H12A—C12—H12B	108.2	H27A—C27—H27C	109.5
C12—C13—C17	119.33 (16)	H27B—C27—H27C	109.5
C12—C13—C14	111.90 (16)	O2B—C28B—H28D	109.5
C17—C13—C14	104.72 (15)	O2B—C28B—H28E	109.5
C12—C13—H13A	106.7	H28D—C28B—H28E	109.5
C17—C13—H13A	106.7	O2B—C28B—H28F	109.5
C14—C13—H13A	106.7	H28D—C28B—H28F	109.5
C15—C14—C13	101.14 (15)	H28E—C28B—H28F	109.5
C15—C14—C18	105.73 (16)	H1W1—O1W—H2W1	111.2
C13—C14—C18	110.52 (15)		
			
C10—C1—C2—C3	−175.99 (17)	C9—C11—C12—C13	−52.3 (2)
C28A—O2A—C3—O1A	−4.2 (16)	C11—C12—C13—C17	179.56 (17)
C28A—O2A—C3—O1B	−52 (11)	C11—C12—C13—C14	56.9 (2)
C28A—O2A—C3—O2B	10.4 (19)	C12—C13—C14—C15	173.67 (15)
C28A—O2A—C3—C2	177.4 (5)	C17—C13—C14—C15	43.02 (18)
C28B—O2B—C3—O1A	74 (6)	C12—C13—C14—C18	62.0 (2)
C28B—O2B—C3—O1B	−4(3)	C17—C13—C14—C18	−68.62 (19)
C28B—O2B—C3—O2A	−8(3)	C12—C13—C14—C8	−62.44 (19)
C28B—O2B—C3—C2	−175.0 (12)	C17—C13—C14—C8	166.90 (15)
C1—C2—C3—O1A	88.7 (12)	C7—C8—C14—C15	−67.8 (2)
C1—C2—C3—O1B	−96.4 (6)	C27—C8—C14—C15	52.1 (2)
C1—C2—C3—O2A	−92.9 (5)	C9—C8—C14—C15	173.92 (16)
C1—C2—C3—O2B	75.3 (14)	C7—C8—C14—C13	178.27 (16)
C25—C4—C5—C6	−129.0 (2)	C27—C8—C14—C13	−61.8 (2)
C26—C4—C5—C6	47.1 (3)	C9—C8—C14—C13	59.96 (19)
C25—C4—C5—C10	101.9 (2)	C7—C8—C14—C18	55.1 (2)
C26—C4—C5—C10	−81.9 (2)	C27—C8—C14—C18	174.98 (16)
C4—C5—C6—C7	170.36 (17)	C9—C8—C14—C18	−63.2 (2)
C10—C5—C6—C7	−58.8 (2)	C13—C14—C15—C16	−36.41 (19)
C5—C6—C7—C8	57.2 (2)	C18—C14—C15—C16	78.84 (19)
C6—C7—C8—C27	69.8 (2)	C8—C14—C15—C16	−154.78 (17)
C6—C7—C8—C9	−51.2 (2)	C14—C15—C16—C17	17.0 (2)
C6—C7—C8—C14	−168.99 (16)	C12—C13—C17—C20	75.7 (2)
C7—C8—C9—C11	−176.10 (14)	C14—C13—C17—C20	−158.18 (17)
C27—C8—C9—C11	64.9 (2)	C12—C13—C17—C16	−158.89 (18)
C14—C8—C9—C11	−56.07 (19)	C14—C13—C17—C16	−32.73 (19)
C7—C8—C9—C10	51.2 (2)	C15—C16—C17—C20	137.46 (18)
C27—C8—C9—C10	−67.7 (2)	C15—C16—C17—C13	9.6 (2)
C14—C8—C9—C10	171.27 (15)	C23—O3—C20—C24	98.95 (19)
C2—C1—C10—C19	172.70 (17)	C23—O3—C20—C17	−138.00 (17)
C2—C1—C10—C9	49.8 (2)	C23—O3—C20—C21	−19.3 (2)
C2—C1—C10—C5	−68.2 (2)	C13—C17—C20—O3	−67.8 (2)
C11—C9—C10—C19	−61.2 (2)	C16—C17—C20—O3	171.40 (15)
C8—C9—C10—C19	70.7 (2)	C13—C17—C20—C24	50.0 (3)
C11—C9—C10—C1	55.2 (2)	C16—C17—C20—C24	−70.9 (3)
C8—C9—C10—C1	−172.91 (15)	C13—C17—C20—C21	179.70 (17)
C11—C9—C10—C5	174.77 (15)	C16—C17—C20—C21	58.9 (2)
C8—C9—C10—C5	−53.4 (2)	O3—C20—C21—C22	25.6 (2)
C4—C5—C10—C19	58.6 (2)	C24—C20—C21—C22	−88.4 (2)
C6—C5—C10—C19	−70.8 (2)	C17—C20—C21—C22	140.6 (2)
C4—C5—C10—C1	−55.6 (2)	C20—C21—C22—C23	−23.4 (2)
C6—C5—C10—C1	174.97 (15)	C20—O3—C23—O4	−175.35 (18)
C4—C5—C10—C9	−175.60 (16)	C20—O3—C23—C22	4.4 (2)
C6—C5—C10—C9	55.00 (19)	C21—C22—C23—O4	−167.6 (2)
C10—C9—C11—C12	−172.33 (16)	C21—C22—C23—O3	12.6 (2)
C8—C9—C11—C12	53.5 (2)		

### Hydrogen-bond geometry (Å, °)

**Table:**

*D*—H···*A*	*D*—H	H···*A*	*D*···*A*	*D*—H···*A*
O1W—H1W1···O4^i^	1.06	1.94	2.912 (4)	151
C2—H2A···O4^ii^	0.97	2.45	3.305 (3)	146
C12—H12B···O3	0.97	2.58	3.154 (2)	118

Symmetry codes: (i) −*x*, *y*+1/2, −*z*+3/2; (ii) −*x*, *y*−1/2, −*z*+3/2.
